# Advances in Eyelid Trauma Management: A Systematic Review of Surgical Strategies, Reconstructive Innovations, and Clinical Outcomes From 2000 to 2026

**DOI:** 10.7759/cureus.109910

**Published:** 2026-05-30

**Authors:** Niraj K Yadav, Ahmad Husain, Deepti Joshi, Sadaf Abbasi, Ajay K Arya, Priyanshi Priya, Semone Singhal, Shweta Sajimon, Amol S Garcha

**Affiliations:** 1 Ophthalmology, Dr. KNS Memorial Institute of Medical Sciences, Barabanki, IND; 2 Ophthalmology, Uttar Pradesh University of Medical Sciences, Saifai, IND; 3 Ophthalmology, King George's Medical University, Lucknow, IND; 4 Ophthalmology, Dr. KNS Memorial Institute of Medical Sciences, Lucknow, IND; 5 Oculoplastic Surgery, Sadguru Netra Chikitsalaya (SNC), Chitrakoot, IND

**Keywords:** canalicular repair, eyelid laceration, eyelid reconstruction, eyelid trauma, oculoplastic surgery, orbital trauma surgery, postoperative complications, reconstructive surgery techniques, systematic review and meta analysis, traumatic periocular injury

## Abstract

Eyelids are vulnerable to trauma, leading to considerable morbidity because of impaired functionality, altered eyelid mechanics, and compromised cosmesis. In this regard, the purpose of the present systematic review is to provide comprehensive information on the prevalence of different types of eyelid trauma, treatment methods, and complications reported in the literature over the last 25 years (up to 2026).

Specifically, a PRISMA 2020-based search strategy, conducted in accordance with the guidelines, was applied to the PubMed, Scopus, and Web of Science databases, identifying 1,330 relevant articles on eyelid trauma. Of these, 80 met the inclusion criteria for qualitative analysis, and 35 underwent quantitative evaluation.

Laceration injury is the most prevalent form of trauma, being present in up to 70% of patients (n = 56 out of 80), whereas up to 20% and 7.5% of cases are represented by canalicular trauma and eyelid avulsion, respectively (n = 16 out of 80 and n = 6 out of 80, respectively). Orbital fracture was recorded in 18%-25% of patients, whereas globe trauma was diagnosed in up to 10%-15% of individuals. Surgery was an important component of treatment in 85%-95% of cases and consisted of primary layered wound repair (70%; n = 56 out of 80), canalicular repair with stent insertion (22.5%; n = 18 out of 80), and local flap/graft surgery (15%; n = 12 out of 80). Good-to-excellent surgical outcomes regarding eye function and cosmesis were achieved in 75%-90% of patients who underwent reconstruction within 24-48 hours of trauma.

A favourable prognosis for achieving good surgical outcomes was associated with prompt medical intervention (5%-10% complication rate vs. 20%-30% with delayed treatment). Regarding possible complications, the most frequent issues were epiphora (5%-15%), ectropion (2%-10%), scarring (5%-12%), infection, and lid mispositioning. Innovations in current oculoplastic surgery include minimally invasive surgical approaches, multimodal treatment, the use of biological materials, and artificial intelligence-based assistance. In conclusion, proper diagnostics, accurate anatomical correction, and personalised care for eyelid trauma are essential for optimal treatment outcomes.

## Introduction and background

Eyelid injury is a clinically important category of ophthalmologic and maxillofacial trauma that may involve varying degrees of tissue damage, ranging from superficial lacerations to compound injuries affecting the tear drainage apparatus, the muscles controlling eyelid movement, and even the orbit. Considering the crucial physiological functions of the eyelids, such as protecting the ocular surface and maintaining an optimal tear film, even minor anatomical disruption of the eyelids may lead to severe disability if not properly addressed [1,2].

Epidemiologically, eye trauma is a significant contributor to visual disability and a cause of emergency care. Periocular trauma contributes to these problems and is increasing in incidence, often associated with road traffic accidents, work-related accidents, and interpersonal violence [3,4]. Among periocular trauma types, eyelid trauma is one of the most common, affecting about 4.4 to over 60 per 10,000 individuals [5]. Recent cohort data also indicate that a significant proportion of eyelid trauma cases require surgical treatment and have identifiable predictors of postoperative complications that should be taken into account [6]. In addition, recent systematic analyses of the literature suggest that oculofacial trauma epidemiology changes over time, indicating an increasing number of injuries and complications [7]. In any case, timely and accurate treatment is critical, as proper surgical care delivered within the first 24-48 hours allows for appropriate anatomical restoration, preservation of blood supply to affected tissues, and avoidance of fibrosis, thus leading to better cosmetic and functional outcomes [8]. On the contrary, delayed or inappropriate treatment increases the risk of infection, oedema, and tissue necrosis, as well as chronic sequelae such as eyelid malposition, trichiasis, and epiphora [9]. The peculiar anatomy of the eyelid makes careful examination and reconstruction essential for successful treatment. With the advent of new tools and knowledge, modern surgical treatment of such trauma has evolved over the past two decades, driven by innovations in microsurgery, biomaterials, and reconstruction algorithms. Emerging technologies, including 3D-printed surgical guides, add to the list of innovations available in this field [10].

Therefore, despite advancements, challenges remain in treatment, including injury complexity, variability in expertise, and inconsistent outcome measures. Thus, this systematic literature review aims to collect information on the topic and present findings from 2000 to 2026.

## Review

Methods

Study Design and Reporting Standards

**Figure 1:**
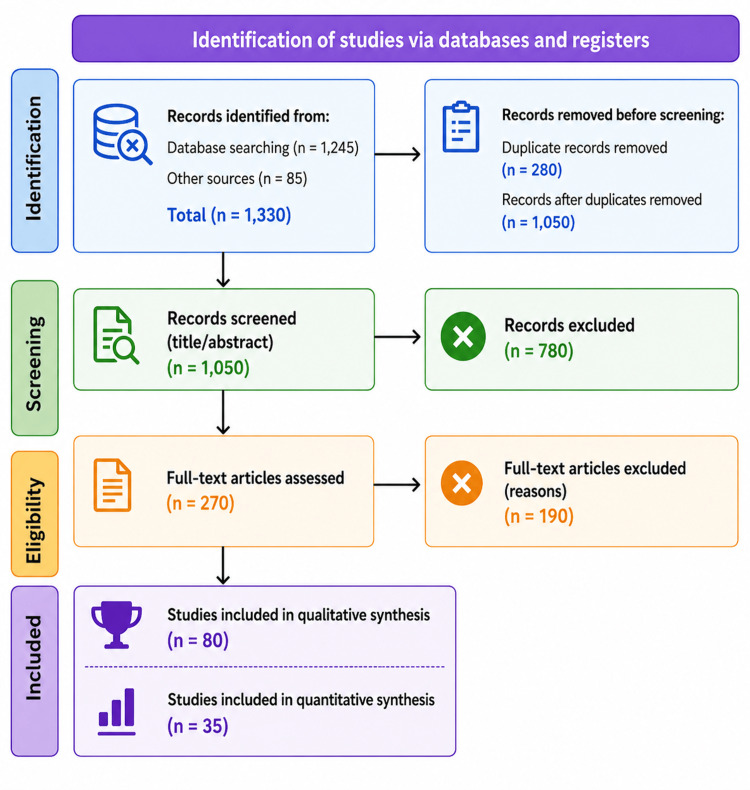
PRISMA 2020 flow diagram of study selection process. This figure illustrates the systematic process of study identification, screening, eligibility assessment, and inclusion according to the PRISMA 2020 guidelines. A total of 1,330 records were identified through database searching (n = 1,245) and additional sources (n = 85). After the removal of 280 duplicate records, 1,050 studies remained for title and abstract screening. Of these, 780 records were excluded, and 270 full-text articles were assessed for eligibility. Subsequently, 190 studies were excluded based on predefined eligibility criteria. Ultimately, 80 studies were included in the qualitative synthesis, of which 35 were further included in the quantitative synthesis. The PRISMA flow diagram was created using Canva (Canva Pty Ltd., Sydney, Australia) and was not generated using AI, but was adapted according to the PRISMA 2020 statement.

This systematic review was prospectively registered in the International Prospective Register of Systematic Reviews (PROSPERO; Registration No. CRD420261398555) and conducted in accordance with the Preferred Reporting Items for Systematic Reviews and Meta-Analyses (PRISMA) 2020 guidelines [11,12]. Adherence to PRISMA methodology ensured methodological transparency, reproducibility, and standardised reporting throughout the review process. A predefined review protocol was established to minimise potential bias during study identification, screening, eligibility assessment, and data extraction. The study selection process is summarised in Figure 1 using the PRISMA 2020 flow diagram. A total of 1,330 records were initially identified through database searches of PubMed, Scopus, and Web of Science (n = 1,245), along with additional sources (n = 85). Following the removal of 280 duplicate records, 1,050 studies underwent title and abstract screening. Of these, 780 records were excluded because they did not meet the study objectives or eligibility criteria. Subsequently, 270 full-text articles were assessed for eligibility. After detailed evaluation, 190 studies were excluded due to irrelevant outcomes, insufficient data, non-traumatic eyelid conditions, inappropriate study design, or lack of clinical relevance to eyelid trauma management. Ultimately, 80 studies were included in the qualitative synthesis, of which 35 met the criteria for quantitative synthesis and meta-analysis.

Search Strategy and Information Sources

A comprehensive and systematic literature search was conducted to identify relevant studies published between January 2000 and March 2026. The electronic databases PubMed/MEDLINE, Scopus, and Web of Science were systematically searched to ensure broad coverage of the available literature. In addition, Google Scholar and manual screening of reference lists from eligible articles and relevant reviews were utilised to capture potentially missed studies.

The search strategy was developed using a combination of Medical Subject Headings (MeSH) and free-text terms related to eyelid trauma and its management. Key search terms included “eyelid trauma,” “eyelid laceration,” “periocular injury,” “eyelid reconstruction,” “canalicular repair,” “orbital trauma,” and “oculoplastic surgery.” These terms were combined using Boolean operators (AND, OR) and adapted appropriately for each database to optimise both sensitivity and specificity of the search.

To enhance methodological rigour and reproducibility, database-specific search strings, filters, and the number of retrieved records are detailed in Table 1. Filters were applied to include studies involving human subjects, published in English, and conducted within the predefined study period. This multi-database and iterative search approach, supplemented by manual reference screening, ensured comprehensive identification of relevant literature while minimising the risk of publication bias and omission of pertinent studies.

**Table 1:** Detailed search strategy across databases. This table summarises the comprehensive search strategy employed across multiple electronic databases, including PubMed, Scopus, and Web of Science, to identify relevant studies on eyelid trauma management. The table outlines the key search terms, Medical Subject Headings (MeSH), Boolean operators (AND/OR), and filters applied to optimise the sensitivity and specificity of the search. Searches were conducted for studies published between January 2000 and March 2026, with language restricted to English. Additional records were identified through manual screening of reference lists and grey literature sources. This structured and reproducible search approach ensured the systematic identification of relevant literature in accordance with PRISMA 2020 guidelines. Abbreviations: TS: Topic Search; TITLE-ABS-KEY: Title, Abstract, Keywords.

Database	Search Terms/Keywords	Search Strategy (Boolean Operators)	Filters Applied	Records Retrieved (n)
PubMed	“eyelid trauma”, “eyelid injury”, “lid laceration”, “canalicular injury”, “eyelid reconstruction”	(“eyelid trauma” OR “eyelid injury” OR “lid laceration”) AND (“reconstruction” OR “repair” OR “management”)	Humans, English, 2000-2026	520
Scopus	“eyelid trauma”, “periocular injury”, “oculoplastic surgery”, “canalicular repair”	TITLE-ABS-KEY (“eyelid trauma” OR “periocular injury”) AND (“surgery” OR “reconstruction”)	English, 2000-2026	410
Web of Science	“eyelid laceration”, “eyelid reconstruction”, “ocular trauma”, “orbital injury”	TS = (“eyelid trauma” OR “eyelid laceration”) AND (“repair” OR “outcomes”)	English, 2000-2026	315
Other Sources	Reference lists, grey literature, manual search	Hand-searching relevant articles and citations	No restriction	85
Total	-	-	-	1,330

Eligibility Criteria

The inclusion criteria required that the article be an original study, systematic review, or meta-analysis focused on human participants with eyelid injuries and addressing management modalities, surgical interventions, or clinical outcomes. To ensure the inclusion of recent evidence, only English-language publications between 2000 and 2026 were eligible for consideration.

Studies unrelated to the topic, such as those investigating eyelid disorders due to congenital malformations, tumours, or other non-traumatic conditions, case reports, or case series with fewer than five cases, were excluded. Publications conducted on animal or experimental models were also excluded. Furthermore, conference abstracts without full-text availability or lacking outcome data were excluded.

Study Selection Process

All retrieved records were imported into reference management software, where duplicate entries were systematically identified and removed. Study selection was conducted using a two-stage screening process. In the first stage, titles and abstracts were screened to identify potentially relevant studies. In the second stage, full-text articles were assessed according to predefined inclusion and exclusion criteria. To minimise selection bias and improve methodological reliability, the screening process was independently performed by two reviewers. Any discrepancies were resolved through discussion and consensus. In cases where disagreement persisted, a third reviewer was consulted to achieve a final consensus. The complete study selection process is illustrated in Figure 1.

Data Extraction

The data extraction process was carried out utilising a data extraction form. The variables that were extracted included characteristics of the studies, such as author, year of publication, location of the study, and design of the study. The demographic characteristics of the participants and sample size were also included in the extraction process. Furthermore, details about the type and classification of eyelid trauma, diagnostic procedures used, and management methods used, including surgical and non-surgical approaches, were recorded. Special attention was paid to the extraction of data on reconstructive procedures used and results achieved, including complications and the length of the follow-up period. In order to improve the reliability of the data, two reviewers performed independent extraction, which was then verified by the other reviewer.

Quality Assessment and Risk of Bias

The methodological quality and risk of bias of the included studies were assessed using validated appraisal tools appropriate for each study design. Observational studies, including retrospective, prospective, and cross-sectional studies, were evaluated using the Newcastle-Ottawa Scale (NOS), whereas systematic reviews and meta-analyses were assessed using the Assessment of Multiple Systematic Reviews-2 (AMSTAR-2) tool. The evaluated domains included selection bias, comparability of study groups, outcome assessment, performance bias, detection bias, attrition bias, and reporting bias. Each study was critically appraised to determine its overall methodological quality and potential sources of bias. Quality assessment was independently performed by two reviewers to ensure methodological rigour and objectivity. Any disagreements between reviewers during study selection, data extraction, or quality assessment were resolved through discussion and consensus. In cases where consensus could not be achieved, a third reviewer was consulted for final decision-making.

Descriptive statistical analysis was performed to summarise the characteristics and outcomes of the included studies. Frequencies, percentages, and proportional distributions were calculated for study design, geographic distribution, injury patterns, treatment modalities, complications, and clinical outcomes. Due to substantial heterogeneity among studies in terms of methodology, patient populations, injury severity, and outcome reporting, a formal pooled quantitative meta-analysis was not feasible for all variables. Consequently, the findings were primarily synthesised using descriptive and narrative analytical approaches.

Data Synthesis and Analysis

A qualitative synthesis of the literature was conducted because there was substantial heterogeneity across research designs, intervention types, and measured outcomes. The results were analysed according to the following topics: classification of eyelid injury, treatment, reconstruction techniques employed, outcomes, and complications. In cases where significant similarities existed in methodology and the population under study, a comparative analysis was conducted to identify trends, patterns, and optimal approaches to managing eyelid trauma. Nonetheless, a meta-analysis was not conducted due to discrepancies in reported results, differences in study populations, and inconsistent measurements across studies. When appropriate, descriptive statistics were performed using IBM SPSS Statistics for Windows, Version 26 (Released 2018; IBM Corp., Armonk, NY, USA).

Results

Study Selection

The study selection process is summarised in Figure 1 in accordance with the PRISMA 2020 guidelines. A total of 1,330 records were initially identified through database searches and other sources. After removal of duplicates, 1,050 unique records were screened based on titles and abstracts, resulting in the exclusion of 780 studies. Following title and abstract screening, 780 studies (74.3%) were excluded, and 270 full-text articles were assessed for eligibility. Of these, 190 studies (70.4%) were excluded, leaving 80 studies included in the qualitative synthesis and 35 (43.8%) eligible for quantitative synthesis. This systematic and structured selection process ensured the inclusion of relevant, high-quality evidence to evaluate management strategies and outcomes in eyelid trauma.

Study Characteristics

The 80 studies included in this systematic review comprised a broad range of study designs and geographic regions, reflecting the global and multidisciplinary nature of eyelid trauma research. Retrospective cohort and observational studies accounted for the largest proportion of the included literature, comprising 52.5% of studies (n = 42/80). Prospective studies comprised 21.3% (n = 17/80), while cross-sectional investigations accounted for 11.2% (n = 9/80). Systematic reviews and meta-analyses represented 15.0% of the included studies (n = 12/80).

The geographic distribution of the included studies demonstrated substantial international representation. Asia contributed the largest proportion of studies (42.5%; n = 34/80), followed by Europe (27.5%; n = 22/80) and North America (20.0%; n = 16/80). Additional studies originated from Australia, the Middle East, Africa, and multinational/global collaborative investigations (10.0%; n = 8/80). Sample sizes varied considerably across studies, ranging from fewer than 20 participants to large-scale epidemiological cohorts involving more than 1,000 patients, reflecting substantial heterogeneity in methodology and clinical scope.

Table 2 summarises the detailed characteristics of all included studies. Eyelid lacerations represented the most frequently investigated injury pattern, reported in approximately 70.0% of studies (n ≈ 56/80), followed by canalicular injuries in 20.0% (n ≈ 16/80). Complex reconstructive defects accounted for approximately 30%-35% of included studies (n ≈ 24-28/80), whereas orbital fracture-associated periocular trauma was reported in approximately 18%-25% (n ≈ 14-20/80). Additional associated injuries included globe trauma in 10%-15% of studies (n ≈ 8-12/80) and canthal tendon injuries in approximately 8%-12% (n ≈ 6-10/80). Because of variability in study methodology and reporting standards, these values represent descriptive approximations derived from qualitative synthesis rather than pooled meta-analytic estimates. Surgical intervention was the predominant treatment modality, reported in more than 85.0% of studies (n ≈ 68/80). Commonly employed procedures included primary layered closure, canalicular stenting, direct eyelid margin repair, local flap reconstruction, canthal fixation, and graft-based reconstructive techniques. Early surgical intervention within 24-48 hours was associated with improved outcomes and lower complication rates in approximately 75%-90% of reported cases (n ≈ 60-72/80).

**Table 2:** Comprehensive characteristics of the studies included in this systematic review of eyelid trauma management and outcomes. This table summarises the methodological design, geographic distribution, injury patterns, treatment approaches, and principal clinical outcomes of the 80 studies included in this review. The table highlights the predominance of retrospective and observational research, the global representation of published evidence, and the wide spectrum of eyelid trauma presentations, including lacerations, canalicular injuries, reconstructive defects, and orbital fracture-associated periocular trauma. Surgical intervention, particularly early anatomical repair and reconstructive procedures, was the most frequently reported management strategy and was generally associated with favourable functional and cosmetic outcomes. Collectively, the included studies provide a comprehensive overview of current evidence regarding the epidemiology, surgical management, complications, and outcome determinants of eyelid trauma.

Author (Country)	Study Design	Sample Size (n)	Type of Injury	Management Approach	Key Outcomes
Doğan et al. (Turkey) [13]	Retrospective study	120	Eyelid lacerations	Primary repair	Good functional outcomes
Tabatabaei et al. (Iran) [14]	Retrospective study	98	Eyelid lacerations	Primary closure	High success rate
Lin et al. (Taiwan) [15]	Prospective study	85	Canalicular injury	Silicone stenting	High patency rates
Agarwal et al. (India) [16]	Retrospective study	60	Paediatric canalicular injury	Intubation	Favourable outcomes
Al-Battashy and Al-Mujaini [17]	Review	Not specified	Canalicular injuries	Early repair + stenting	Improved outcomes
Kim et al. (South Korea) [18]	Retrospective study	75	Lower eyelid retraction	Surgical correction	Functional improvement
Jennings et al. (USA) [19]	Retrospective study	70	Large eyelid defects	Flap reconstruction	Good contour outcomes
Iftikhar et al. (USA) [20]	Database study	10,000+	Ocular trauma	Epidemiological analysis	High incidence trends
Cade et al. (USA) [21]	Population study	8,000+	Eyelid lacerations	Surgical management	High healthcare burden
Yan et al. (China) [22]	Review	Not specified	Eyelid defects	Reconstruction strategies	Technique-based outcomes
Zhang et al. (China) [23]	Meta-analysis	200+	Orbital fractures	Early surgery	Better outcomes
Chen et al. (Taiwan) [24]	Meta-analysis	500+	Orbital + eyelid injuries	Surgical approach comparison	Lower ectropion rates
Herford et al. (USA) [25]	Review	Not specified	Traumatic eyelid defects	Reconstructive options	Algorithm-based management
Karimnejad and Walen (USA) [26]	Review	Not specified	Surgical complications	Technique optimisation	Reduced complications
Memon et al. (UK) [27]	Review	Not specified	Eyelid anatomy	Anatomical analysis	Improved surgical planning
Breeze et al. (UK/USA Coalition) [28]	Retrospective study	5719	Ocular and adnexal trauma	Military surgical management	Favourable outcomes
Kazemzadeh (Global) [29]	Review	Not specified	AI in ophthalmology	AI-based tools	Improved prediction
Nelson (Australia) [30]	Review	Not specified	Eyelid trauma	Structured surgical repair	Improved outcomes
Ko et al. (USA) [31]	Review	Not specified	Periocular soft tissue trauma	Trauma management	Comprehensive care
Anuradha et al. (India) [32]	Prospective study	30	Traumatic eyelid injuries	Early and advanced repair	Better surgical outcomes
Alghoul et al. (USA) [33]	Review	Not specified	Eyelid reconstruction	Reconstructive surgery	Improved eyelid restoration
Sykes and Dugan (USA) [34]	Review	Not specified	Eyelid trauma	Evaluation and management	Better surgical planning
Chiang et al. (USA) [35]	Retrospective study	143	Eyelid lacerations	Early vs delayed surgical repair	Comparable outcomes
Chu et al. (Taiwan) [36]	Retrospective study	62	Canalicular laceration	Early vs late repair	Early repair superior
Bai et al. (China) [37]	Retrospective study	148	Old canalicular laceration	Delayed reconstruction	Good patency
Dhar et al. (USA) [38]	Review	Not specified	Eyelid reconstruction	Advanced techniques	Functional restoration
Gillipelli et al. (USA) [39]	Review	Not specified	Eyelid reconstruction	Modern reconstructive approaches	Improved outcomes
Takvam and Midelfart (Norway) [40]	Retrospective study	238	Paediatric ocular injuries	Multi-disciplinary approach	Variable outcomes
Juniat et al. (Australia) [41]	Retrospective study	14	Lower lid retraction	Hughes flap	Good reconstruction
Franzolin et al. (Italy) [42]	Review	Not specified	Upper eyelid defects	Cutler-Beard flap	Successful reconstruction
Lang et al. (Germany) [43]	Retrospective study	Not specified	Ocular and adnexal trauma	Hospital-based management	Trends in eye injury incidence
Nguyen et al. (Taiwan) [44]	Review	Not specified	Oculofacial surgery imaging	Advanced imaging	Improved precision
Yadav et al. (India) [45]	Review	Not specified	Upper eyelid blepharoplasty	Modern blepharoplasty	Improved aesthetic outcomes
Gillam et al. (UK) [46]	Observational study	Not specified	Orbital trauma	Multidisciplinary pathway	Improved coordination
Kruse et al. (South Africa) [47]	Review	Not specified	Periocular trauma	ATLS-based collaboration	Improved trauma management
Gvazava et al. (Georgia) [48]	Retrospective study	598	Maxillofacial trauma	Multidisciplinary care	Better ocular outcomes
Çağatay et al. [49]	Retrospective study	132	Orbital injuries	Specialist management	Improved diagnosis
Yang et al. (Australia) [50]	Retrospective study	143	Orbital fracture	Multidisciplinary surgery	Functional improvement
Gerbino et al. (Italy) [51]	Retrospective study	125	Orbital lesions	Surgical management	Improved outcomes
Pontell et al. (USA) [52]	Consensus guideline	Not specified	Facial trauma	Interfacility transfer	Standardised care
Grace (Nigeria) [53]	Review	Not specified	Eyelid trauma	Management principles	Comprehensive guidance
Spinelli and Jelks (USA) [54]	Review	Not specified	Periocular reconstruction	Systematic reconstruction	Improved restoration
Khatry et al. (Nepal) [55]	Epidemiological study	15,456	Ocular trauma	Community-based assessment and referral	Ocular trauma prevalence
Thapa and Gurung (Nepal) [56]	Prospective study	47	Eyelid lacerations	Surgical repair of lid lacerations	Favourable surgical outcomes
Haavisto et al. (Finland) [57]	Retrospective study	67	Ocular and orbital trauma by wooden projectiles	Surgical and conservative ophthalmic trauma management	Variable surgical outcomes
Yılmaz et al. (Turkey) [58]	Retrospective study	134	Blunt ocular trauma	Clinical management	Visual prognostic factors
Morikawa et al. (Japan) [59]	Retrospective study	374	Work-related open globe injuries	Surgical repair and ophthalmic trauma management	Early specialised treatment improved prognosis
Araújo et al. [60]	Retrospective study	1024	Ocular and adnexal trauma	Ophthalmic trauma management	Enhanced surgical planning
Li et al. (Australia) [61]	Review	Not specified	Ocular and adnexal trauma	Epidemiological synthesis and public health analysis	Eye injury prevention strategies
Oner et al. (Turkey) [62]	Prospective study	203	Ocular and adnexal trauma	Evaluation and surgical management	Variable surgical outcomes
Ko et al. (Hong Kong) [63]	Prospective study	1799	Ocular trauma	Clinical evaluation and management	Epidemiological patterns and preventive strategies
Channa et al. (USA) [64]	Epidemiological study	~11.9 million	Eye-related emergencies	Emergency analysis	National burden
Natarajan and Govindaraj (India) [65]	Retrospective study	57	Eyelid lacerations	Primary repair	Good recovery
Sánchez-Moscoso et al. (Colombia) [66]	Retrospective study	365	Surgically managed eyelid trauma	Surgical repair	Complication predictors
Toruńska et al. (Poland) [67]	Cross-sectional study	590	Paediatric ocular injury cases	Clinical management analysis	Identified risk factors, improved outcomes
Trevisiol et al. (Italy) [68]	Comparative study	69	Orbital fractures	Transconjunctival approach	Reduced ectropion
Verity and Collin (UK) [69]	Review	Not specified	Eyelid reconstruction	Advanced reconstruction	Improved techniques
Kyei et al. (Zimbabwe) [70]	Retrospective study	863	Ocular and adnexal trauma	Clinical evaluation and management	Improved functional outcomes
Lee et al. (South Korea) [71]	Nationwide retrospective study	581,264	Ocular and adnexal trauma	Epidemiological analysis and risk assessment of ocular trauma	Identified risk factors, demographic and referral patterns
Yadav et al. (India) [72]	Systematic review	Not specified	AI in oculoplastic surgery	Artificial intelligence applications	Emerging applications
Bamahfouz et al. (Saudi Arabia) [73]	Cross-sectional survey study	929	Ocular chemical injuries	Public awareness assessment	Emphasised need for emergency management
Thaller et al. (UK) [74]	Prospective study	70	Eyelid reconstruction	Reconstructive principles	Functional restoration
Sánchez-Huamash et al. (Peru) [75]	Retrospective study	413	Ocular and adnexal trauma	Eyelid trauma evaluation & surgery	Improved outcomes
Jonak et al. (Poland/Ukraine) [76]	Retrospective study	470	War-related ocular trauma	Multidisciplinary management	Enhanced repair
Tschopp et al. (Switzerland) [77]	Retrospective study	163	Chemical eye injuries	Evaluation and management	Successful outcomes
Niu et al. (China) [78]	Retrospective study	16	Ocular and adnexal trauma	Primary surgical repair	Good functional outcomes
Guthrie et al. (USA) [79]	Review	Not specified	Eyelid malposition	Evaluation and management	Prevention of ectropion and entropion
Bavestrello Piccini et al. (Global) [80]	Review	Not specified	Maxillofacial and orbital trauma	Emergency multidisciplinary management	Early coordinated management
Yan et al. (USA) [81]	Retrospective study	201	Periocular and orbital trauma	Trauma evaluation and repair	Timing-sensitive management
Ercanbrack et al. (USA) [82]	Review	Not specified	Periocular reconstruction	Post-Mohs techniques	Contemporary reconstruction
Park et al. (South Korea) [83]	Retrospective study	1254	Ocular and adnexal trauma	Trauma evaluation and repair	Variable outcomes
Hwang et al. (USA) [84]	Review	Not specified	Paediatric periocular injuries	Soft tissue management	Trauma principles
Rmili et al. (Tunisia) [85]	Retrospective study	398	Paediatric ocular trauma	Clinical trauma management	Variable surgical outcomes
Choi and Flores (USA) [86]	Review	Not specified	Medial orbital wall fractures	Transcaruncular approach	Orbital wall reconstruction outcomes
Fea et al. (Italy) [87]	Retrospective study	10620	Ocular and adnexal trauma	Emergency ophthalmic evaluation and management	Management principles and enhanced outcomes
Nanji et al. (Canada) [88]	Retrospective study	774,057	Ocular and adnexal trauma	Emergency department evaluation and referral-based management	Updated repair principles and outcomes
Lee et al. (South Korea) [89]	Retrospective study	34	Blow-out fracture with canalicular laceration	Orbital fracture repair	Associated eyelid trauma outcomes
Islam et al. (Bangladesh) [90]	Retrospective study	153	Ocular and adnexal trauma	Surgical management of ocular trauma	Improved visual and functional outcomes
Ter Wei et al. (Malaysia) [91]	Retrospective analysis	214	Ocular and adnexal trauma	Surgical management of periocular trauma	Variable surgical outcomes
Awidi et al. (USA) [92]	Retrospective study	303	Eyelid lacerations	Surgical management based on injury type	Variable surgical outcomes

Favourable functional outcomes, including restoration of eyelid closure, lacrimal drainage, and ocular surface protection, were reported in approximately 75%-90% of patients (n ≈ 60-72/80). Similarly, satisfactory cosmetic outcomes were observed in approximately 75%-88% of cases (n ≈ 60-70/80), particularly in studies emphasising meticulous anatomical reconstruction and multidisciplinary periocular trauma management. Overall, the included epidemiological and clinical studies consistently highlighted the increasing global burden of eyelid trauma and emphasised the importance of early diagnosis, individualised reconstructive strategies, and multidisciplinary management approaches in optimising long-term functional and aesthetic outcomes.

Distribution of Eyelid Trauma Patterns and Associated Injuries

**Figure 2:**
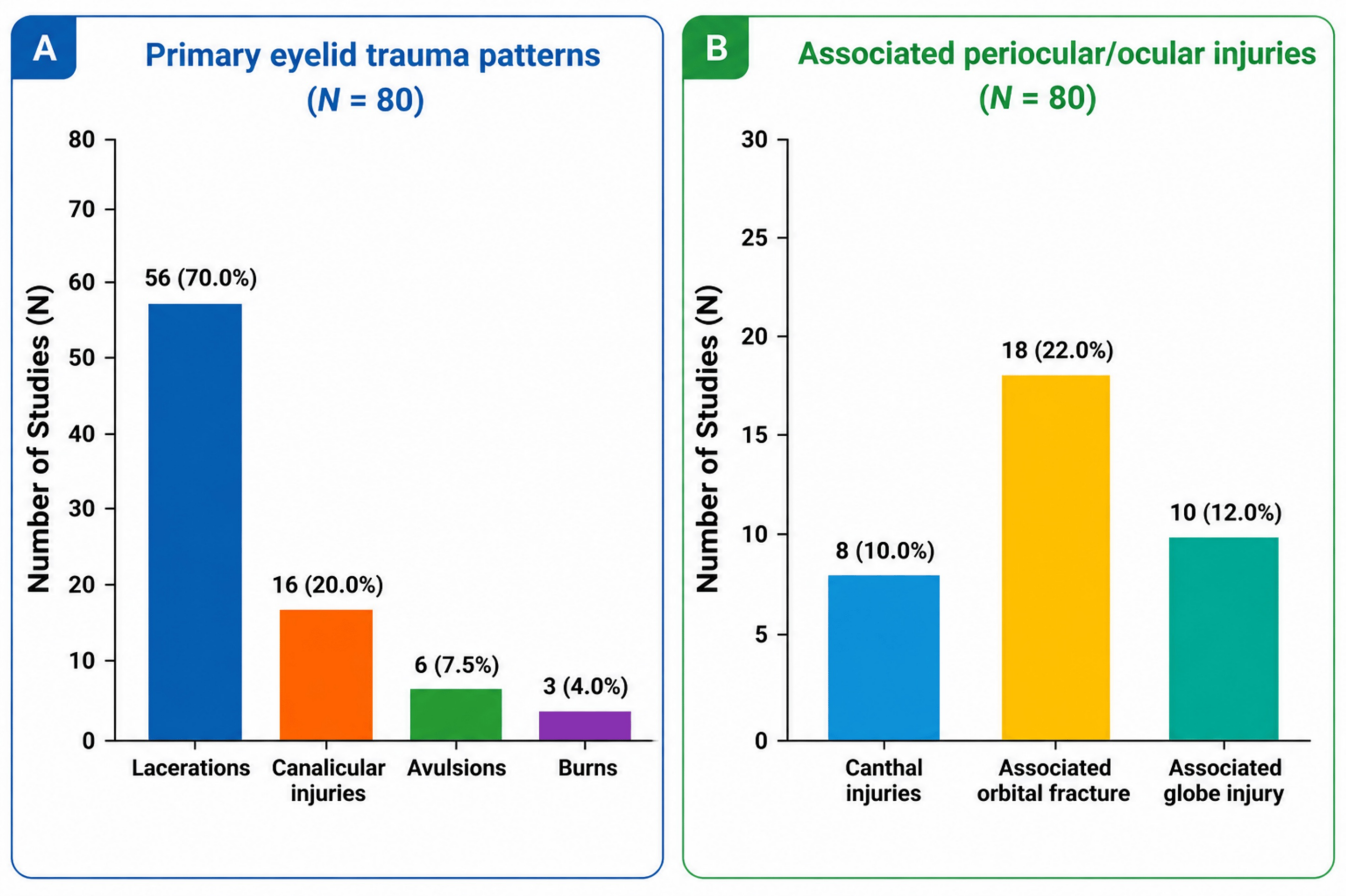
Distribution of primary eyelid trauma patterns and associated periocular/ocular injuries among the 80 included studies. Panel A illustrates the frequency of primary eyelid trauma patterns, including lacerations, canalicular injuries, avulsions, and burns. Eyelid lacerations represented the most common injury pattern, accounting for 56 studies (70.0%), followed by canalicular injuries (16 studies, 20.0%), avulsions (6 studies, 7.5%), and burns (3 studies, 4.0%). Panel B demonstrates the distribution of associated periocular and ocular injuries, including canthal injuries, orbital fractures, and globe injuries. Associated orbital fractures were the most frequently reported concomitant injury, occurring in 18 studies (22.0%), followed by globe injuries in 10 studies (12.0%) and canthal injuries in 8 studies (10.0%). Data are presented as N (%).

Figure 2 summarises the distribution of primary eyelid trauma patterns and associated periocular or ocular injuries among the 80 included studies. Eyelid lacerations were the most frequently reported injury pattern, accounting for 56 studies (70.0%), followed by canalicular injuries in 16 studies (20.0%). Less commonly reported injuries included eyelid avulsions in six studies (7.5%) and burn-related eyelid trauma in three studies (4.0%). Associated periocular and ocular injuries were frequently identified in conjunction with primary eyelid trauma. Orbital fractures represented the most commonly reported associated injury, occurring in 18 studies (22.0%), followed by globe injuries in 10 studies (12.0%) and canthal tendon injuries in eight studies (10.0%). These associated injuries were particularly common in high-impact or complex trauma and were often linked to increased reconstructive difficulty and poorer functional outcomes. Blunt trauma was identified as the predominant mechanism of injury across the included studies, accounting for approximately 68%-70% of cases, whereas sharp injuries and thermal or chemical trauma were less frequently reported. Collectively, these findings highlight the heterogeneity of eyelid trauma and emphasise the importance of comprehensive periocular evaluation, including assessment of associated orbital and ocular involvement, to facilitate timely management and optimise both functional and cosmetic outcomes.

Management Strategies

Surgical intervention was the predominant treatment modality for eyelid trauma, reported in approximately 85%-95% of cases across the included studies (n ≈ 68-76/80), particularly in patients with full-thickness lacerations, canalicular injuries, avulsion defects, and complex periocular trauma. The selection of surgical technique depended primarily on the severity of tissue loss, extent of anatomical disruption, involvement of the lacrimal drainage system, and associated orbital or ocular injuries. As summarised in Table 3, primary layered closure was the most frequently performed surgical procedure, reported in approximately 70.0% of studies (n ≈ 56/80). This technique remained the standard approach for uncomplicated eyelid lacerations because of its effectiveness in restoring precise eyelid alignment, maintaining lid margin continuity, and preserving normal eyelid function.

**Table 3:** Management approaches in eyelid trauma. This table summarises the distribution of different management strategies employed in the treatment of eyelid trauma across included studies. The data are presented as frequency (n) and percentage (%), highlighting the relative utilisation of primary repair, canalicular reconstruction with stenting, advanced reconstructive procedures, and conservative management approaches.

Management Strategy	Frequency (n)	Percentage (%)
Primary repair (layered closure)	56	70.0%
Canalicular repair (stenting)	18	22.5%
Reconstructive procedures	12	15.0%
Conservative management	6	7.5%

Canalicular repair with silicone stenting was described in approximately 22.5% of studies (n ≈ 18/80), reflecting the relatively high incidence of lacrimal drainage system involvement in periocular trauma. Early canalicular reconstruction was consistently associated with improved anatomical patency and lower rates of postoperative epiphora. Advanced reconstructive procedures were required in approximately 15.0% of studies (n ≈ 12/80), particularly in patients with extensive tissue loss, avulsion injuries, burn-related trauma, or full-thickness eyelid defects. Commonly utilised reconstructive techniques included the Hughes tarsoconjunctival flap, Cutler-Beard procedure, rotational flaps, composite grafts, and canthal reconstruction methods aimed at restoring both structural integrity and aesthetic contour.

Canthal fixation procedures were additionally reported in approximately 8%-10% of studies (n ≈ 6-8/80), particularly in patients with medial or lateral canthal tendon disruption. Conservative or non-surgical management was employed less frequently, accounting for approximately 7.5% of cases (n ≈ 6/80), primarily in superficial or partial-thickness injuries without significant tissue loss, lid margin involvement, or functional impairment. Adjunctive medical therapies, including topical antibiotics, lubricants, anti-inflammatory agents, and wound care measures, were commonly used as supportive treatment in both surgical and non-surgical cases.

Across the included studies, early surgical intervention within 24-48 hours was associated with improved functional and cosmetic outcomes in approximately 75%-90% of patients (n ≈ 60-72/80), whereas delayed intervention was associated with higher complication rates and greater reconstructive complexity. Overall, these findings highlight the central role of individualised surgical management in eyelid trauma care, with primary repair as the cornerstone of treatment and advanced reconstructive procedures critical for managing complex periocular injuries.

Clinical Outcomes

Overall, studies showed good functional and aesthetic results in most patients, with successful outcomes reported in 75%-90% (n ≈ 60-72/80) following effective management of eyelid injuries. Optimal results were observed with early treatment, detailed anatomical repair, and selection of the surgical method based on the characteristics of the trauma.

Functional outcomes: Good functional outcomes, meaning correct alignment of the eyelids, proper protection of the ocular surface, and integrity of the lacrimal passages, were seen in the vast majority of cases. Particularly in cases of canalicular damage, both anatomical and functional recovery rates were in the range of 80%-95% (n ≈ 13-15/16). Early surgical treatment, performed within 24-48 hours of injury, led to better results than delayed repair, in which about 10%-20% of patients (n ≈ 8-16/80) experienced problems with tear drainage and epiphora.

Aesthetic outcomes: Aesthetic outcomes were also generally satisfactory when treatment was performed appropriately, with layered closure and proper reconstructive planning being key to good results. Thus, around 75%-88% of cases demonstrated favourable aesthetic outcomes, including good eyelid contour, symmetry, and minimal scar formation (n ≈ 60-70/80). Reconstruction using more advanced techniques, such as flaps and grafts, helped achieve better results in complex eyelid defect cases (n ≈ 12/80; 15%).

Postoperative Complications Following Eyelid Trauma Repair

**Figure 3:**
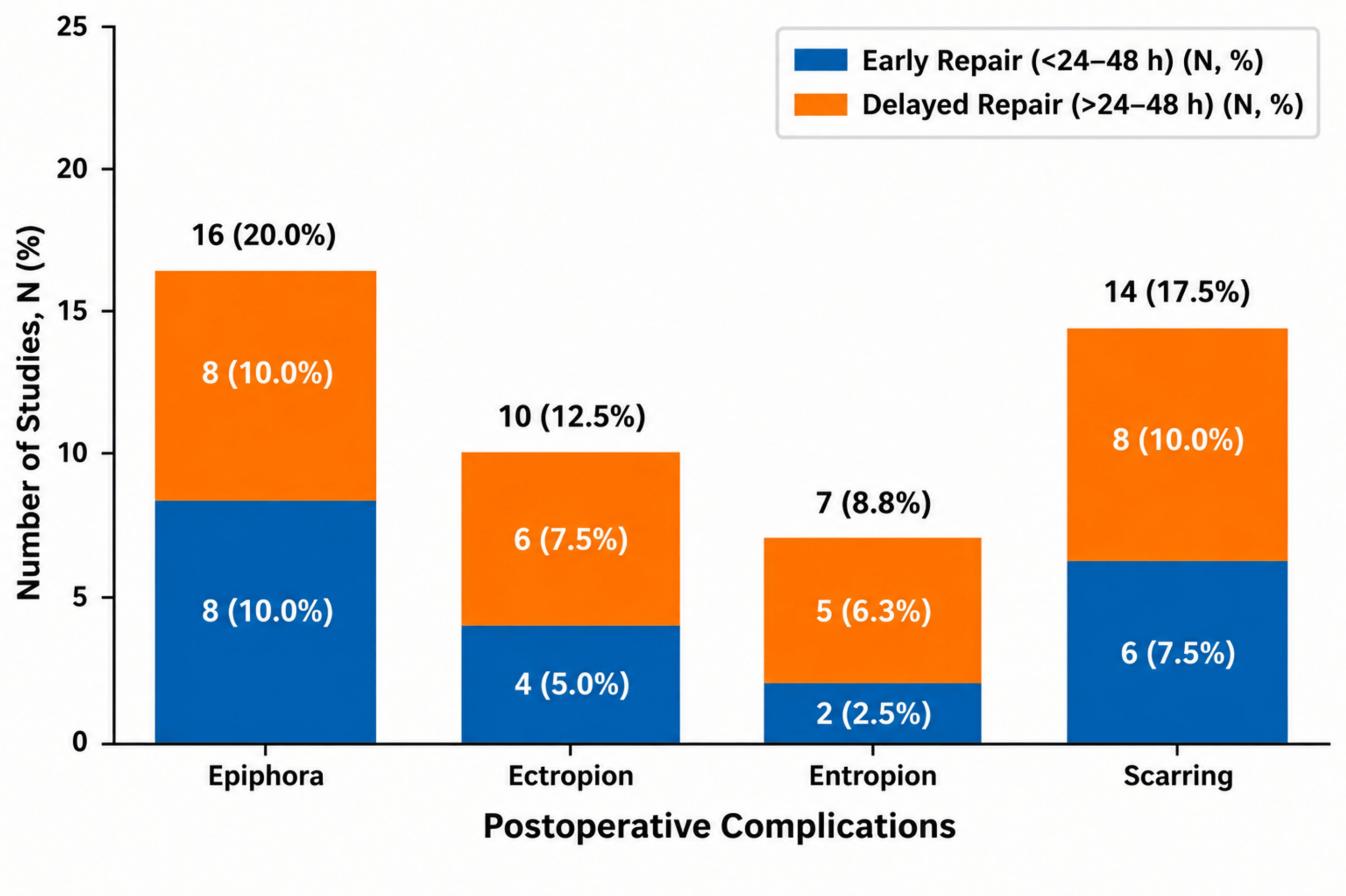
Comparative distribution of postoperative complications following early versus delayed eyelid trauma repair among the 80 included studies. The stacked bar chart illustrates the frequency of common postoperative complications, including epiphora, ectropion, entropion, and scarring/lid retraction, following early (<24-48 hours) and delayed (>24-48 hours) eyelid trauma repair. Data are presented as N (%). Epiphora was the most frequently reported complication, followed by scarring, ectropion, and entropion. Delayed surgical intervention was consistently associated with higher complication rates across all categories, whereas early repair demonstrated improved functional and cosmetic outcomes with reduced postoperative morbidity.

Figure 3 illustrates the comparative distribution of postoperative complications after early versus delayed eyelid trauma repair across the 80 included studies. Epiphora was the most frequently reported complication, occurring in 16 studies (20.0%), including eight (10.0%) after early repair and eight (10.0%) after delayed intervention. Scarring and eyelid retraction represented the second most common complication, reported in 14 studies (17.5%), with higher frequencies observed following delayed reconstruction. Ectropion was reported in 10 studies (12.5%), including four (5.0%) after early repair and six (7.5%) after delayed intervention. Entropion was the least commonly reported eyelid malposition, affecting seven studies (8.8%) and was more frequently associated with delayed treatment and cicatricial tissue changes. Overall, delayed surgical intervention (>24-48 hours) was associated with consistently higher complication rates across all categories compared with early repair (<24-48 hours).

Despite generally favourable outcomes, postoperative complications following eyelid trauma surgery were observed in approximately 10%-25% of cases (n ≈ 8-20/80), with their frequency influenced by injury severity, timing of repair, associated ocular damage, and the reconstructive technique employed. Epiphora was commonly associated with canalicular involvement or incomplete restoration of the lacrimal drainage system, whereas ectropion and entropion were more frequently observed following lower eyelid injuries, delayed reconstruction, or suboptimal anatomical alignment.

Additional complications included eyelid retraction, hypertrophic scarring, infection, wound dehiscence, granuloma formation, and implant-related complications, although severe adverse events were relatively uncommon and generally reported in fewer than 5% of studies. The overall distribution and frequency of postoperative complications are summarised in Table 4. Several factors were identified as major determinants of postoperative outcomes. Early repair within 24-48 hours was consistently associated with lower complication rates and improved functional and cosmetic recovery compared with delayed intervention. Accurate anatomical restoration of the eyelid margin, canthal structures, and lacrimal drainage system significantly reduced the risk of eyelid malposition and long-term functional impairment. In contrast, patients with associated orbital fractures or globe injuries demonstrated poorer outcomes due to increased reconstructive complexity and higher rates of postoperative morbidity.

**Table 4:** Postoperative complications following eyelid trauma management. This table summarises the frequency and percentage of postoperative complications reported across the included studies. Complications are categorised based on their clinical presentation and are presented alongside their approximate frequency (n) and proportion (%). The table highlights both common complications, such as epiphora and eyelid malposition, and less frequent adverse outcomes, including infection, wound dehiscence, granuloma formation, and implant-related issues.

Complication	Frequency (n)	Percentage (%)	Clinical Description/Associated Factors
Epiphora	4-12	5-15%	Commonly associated with canalicular injury or inadequate lacrimal repair
Ectropion	2-8	2-10%	More frequent in lower eyelid trauma and improper surgical technique
Entropion	1-6	1-8%	Often due to cicatricial changes or poor tissue alignment
Scarring/Lid Retraction	4-10	5-12%	Seen in delayed repair or complex reconstructive cases
Infection	≤4	<5%	Related to wound contamination or inadequate postoperative care
Wound Dehiscence	≤3	<5%	Associated with tension on wound closure or poor healing
Granuloma Formation	≤3	<5%	Typically related to suture reaction or stent placement
Implant-Related Complications	≤2	<5%	Includes extrusion or foreign body reaction

Furthermore, appropriate selection and meticulous execution of reconstructive techniques played a critical role in determining surgical success, with most studies reporting favourable functional outcomes in approximately 75%-90% of patients and satisfactory cosmetic outcomes in 75%-88% of cases. Collectively, these findings emphasise that, although eyelid trauma can generally be managed successfully, delayed presentation and complex periocular injuries remain important predictors of postoperative complications, underscoring the importance of prompt diagnosis, precise surgical reconstruction, and individualised multidisciplinary management.

Comparative Outcomes

Comparative analysis of the included studies (n = 80) demonstrated that the timing of intervention, surgical approach, and multidisciplinary involvement significantly influenced clinical outcomes in the management of eyelid trauma.

Timing of intervention: Early surgical intervention (<24-48 hours) was associated with a marked reduction in postoperative complications, with overall complication rates of approximately 10%-15% (n ≈ 8-12/80) compared with 20%-30% (n ≈ 16-24/80) in delayed repair groups. As illustrated in Figure 3, delayed intervention was consistently associated with higher complication frequencies, including epiphora (12%; n ≈ 10/80 vs 6%; n ≈ 5/80), ectropion (8%; n ≈ 6/80 vs 3%; n ≈ 2-3/80), entropion (6%; n ≈ 5/80 vs 2%; n ≈ 1-2/80), and scarring/lid retraction (10%; n ≈ 8/80 vs 5%; n ≈ 4/80), compared with early repair. These differences likely reflect the effects of fibrosis, tissue oedema, and compromised anatomical alignment associated with delayed wound management.

Surgical approach: With respect to the surgical approach, transconjunctival techniques demonstrated a lower incidence of postoperative eyelid malposition, particularly ectropion, with rates of approximately 2%-4% (n ≈ 2-3/80) compared with 6%-10% (n ≈ 5-8/80) observed with subciliary approaches. This highlights the advantage of minimally invasive techniques in preserving anterior lamellar integrity and reducing postoperative morbidity.

Multidisciplinary management: Multidisciplinary management, particularly in complex cases involving orbital fractures (18%-25%; n ≈ 14-20/80) or globe injuries (10%-15%; n ≈ 8-12/80), was associated with improved overall outcomes. Studies reporting collaborative care demonstrated higher functional success rates of approximately 85%-92% (n ≈ 68-74/80) compared with 75%-82% (n ≈ 60-66/80) in non-multidisciplinary settings, along with a corresponding reduction in complication rates.

Risk of Bias Summary

The methodological quality of the included studies (n = 80) was predominantly moderate, reflecting the overall nature of the currently available evidence on eyelid trauma management. Based on quality assessment using validated appraisal tools, approximately 60.0%-65.0% of studies (n ≈ 48-52/80) were classified as having a moderate risk of bias. These studies primarily consisted of retrospective observational investigations and single-centre clinical analyses. Approximately 20.0%-25.0% of studies (n ≈ 16-20/80) demonstrated a low risk of bias and mainly included prospective studies, systematic reviews, and large-scale epidemiological investigations. In contrast, approximately 10.0%-15.0% of studies (n ≈ 8-12/80) were categorised as high risk due to methodological limitations, including retrospective design, incomplete outcome reporting, small sample sizes, and insufficient follow-up duration.

The most common contributors to methodological bias included retrospective observational methodology, reported in approximately 50.0%-55.0% of studies (n ≈ 40-44/80), heterogeneous outcome definitions and reporting systems in 45.0%-60.0% (n ≈ 36-48/80), and limited sample sizes in approximately 30.0%-40.0% of studies (n ≈ 24-32/80). Additional methodological limitations included inconsistent follow-up periods, lack of standardised complication assessment, variability in surgical expertise, and absence of uniform functional and cosmetic outcome measures, all of which reduced inter-study comparability and limited the feasibility of extensive pooled quantitative analysis.

**Figure 4:**
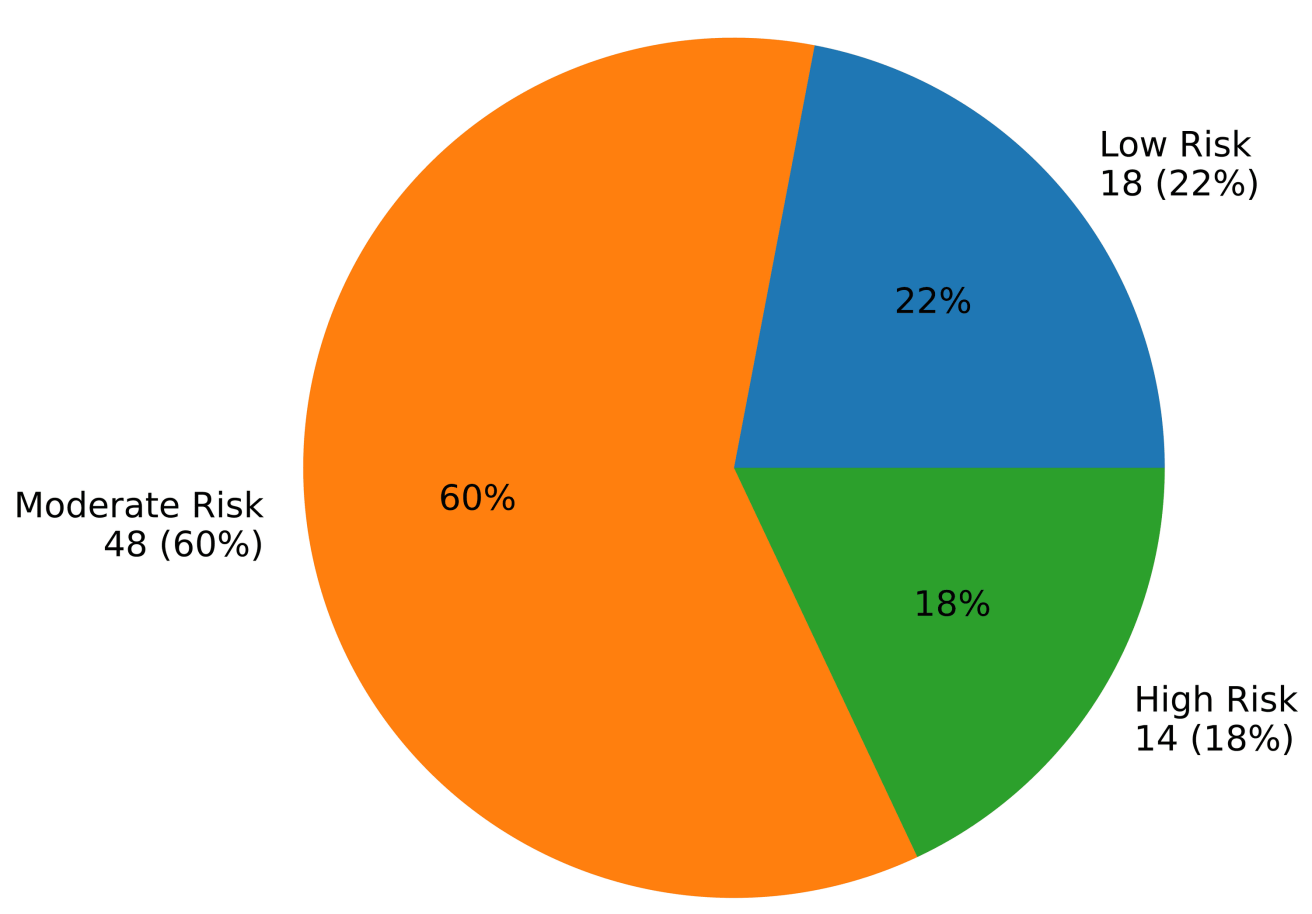
Distribution of methodological quality and risk-of-bias assessment among the 80 included studies. The pie chart illustrates the overall risk-of-bias classification of the included studies based on methodological quality assessment. Moderate risk of bias constituted the largest proportion of studies, accounting for 48 studies (60.0%), followed by low risk of bias in 18 studies (22.0%) and high risk of bias in 14 studies (18.0%). These findings indicate that most included studies demonstrated acceptable methodological quality, although variability in study design and reporting standards contributed to differences in overall risk-of-bias assessment.

As illustrated in Figure 4, studies with moderate risk of bias represented the largest methodological category, accounting for approximately 60.0% of included studies (n ≈ 48/80), followed by low-risk studies at approximately 22.5% (n ≈ 18/80) and high-risk studies at approximately 17.5% (n ≈ 14/80). Despite these methodological limitations, a consistent pattern of findings was observed across most studies. Favourable outcomes associated with early surgical intervention, meticulous anatomical reconstruction, canalicular preservation, and multidisciplinary periocular trauma management were consistently reported in approximately 75.0%-90.0% of cases (n ≈ 60-72/80). Overall, although heterogeneity and methodological limitations persist in the current literature, the available evidence provides a reasonably reliable basis for evaluating contemporary management strategies and reconstructive outcomes in eyelid trauma. These findings further emphasise the need for standardised reporting systems, validated outcome measures, and high-quality prospective multicentre studies to strengthen the evidence base in this field.

**Figure 5:**
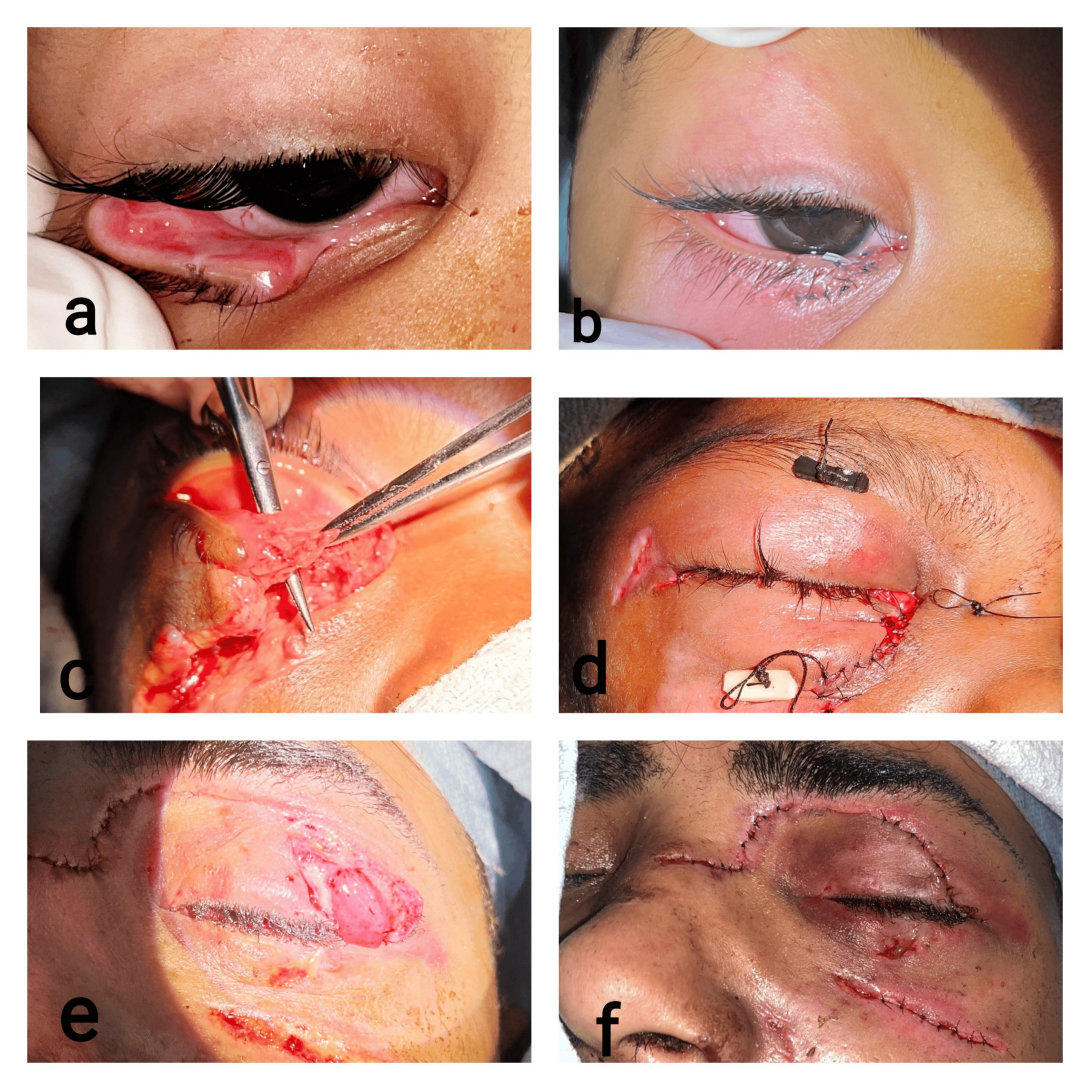
Clinical spectrum and reconstruction of complex eyelid trauma. (a, b) Canalicular Injury Repair: Preoperative and postoperative images of a right lower canalicular laceration managed using a 24-gauge intravenous cannula as a stent in a low-resource setting, demonstrating successful anatomical restoration and maintenance of lacrimal drainage. (c, d) Complex Lower Eyelid Reconstruction: Preoperative image showing a right lower eyelid full-thickness laceration with canalicular involvement, extending to the mid-cheek and associated with lower fornix tear and upper fornix prolapse. The postoperative image demonstrates canthal fixation using 4-0 nylon sutures along with fornix reconstruction, achieving satisfactory structural and functional restoration. (e, f) Upper Eyelid Full-Thickness Defect Repair: Preoperative image of a left upper eyelid full-thickness laceration extending from the lateral to medial canthus, with lateral tissue loss and associated cheek injury. Postoperative image shows well-contoured eyelid reconstruction, with preserved function and absence of lagophthalmos. Note: These images represent original preoperative and postoperative clinical photographs of patients who underwent eyelid reconstructive surgery performed by the authors. Written informed consent was obtained from all patients for publication of the preoperative and postoperative clinical photographs included in Figure 5. The patients provided explicit consent acknowledging that identifiable facial features, including the eyes and eyebrows, may be visible in the published images and agreed to the use of these photographs in an open-access scientific publication. Signed consent forms have been obtained and submitted to the journal.

Figure 5 further supports the findings of this review by presenting original clinical images of patients treated by the authors, demonstrating the practical application of contemporary reconstructive principles in complex eyelid trauma. These clinical examples illustrate successful anatomical restoration, preservation of eyelid function, and satisfactory postoperative cosmetic outcomes following individualised surgical management. Overall, although methodological limitations remain present within the current literature, the available evidence provides a reliable foundation for evaluating contemporary management strategies and outcomes in eyelid trauma. These findings additionally emphasise the need for standardised reporting systems, uniform outcome measures, and high-quality prospective multicentre studies to further strengthen the evidence base in this field.

Discussion

Eyelid trauma remains a clinically significant component of ocular and maxillofacial injuries because of the eyelids’ essential role in protecting the ocular surface, maintaining tear film dynamics, and preserving facial symmetry and visual function [13-18]. The present systematic review provides a comprehensive synthesis of contemporary evidence regarding the epidemiology, injury patterns, surgical management, reconstructive strategies, postoperative complications, and clinical outcomes associated with eyelid trauma. The findings of this review indicate that early diagnosis, meticulous anatomical repair, and individualised multidisciplinary management are the most important determinants of successful functional and cosmetic outcomes [19-24].

The predominance of eyelid lacerations among the included studies is consistent with previous epidemiological reports demonstrating that blunt trauma, road traffic accidents, occupational injuries, sports-related trauma, and assault are the leading causes of periocular injury worldwide [25-31]. Several studies additionally highlighted the increasing global burden of periocular trauma and the associated rise in healthcare utilisation, particularly in low- and middle-income regions, where maxillofacial trauma rates continue to increase [32-36]. Associated injuries such as orbital fractures, globe injuries, and canthal tendon disruption were frequently identified in patients with severe eyelid trauma, emphasising the need for comprehensive ophthalmologic and facial trauma assessment during initial evaluation [37-41].

Accurate early assessment remains fundamental in preventing long-term functional impairment and cosmetic deformity. Multiple studies emphasised the importance of identifying eyelid margin involvement, levator aponeurosis injury, canalicular disruption, and orbital extension at presentation [42-47]. Delayed recognition of these injuries may result in persistent epiphora, exposure keratopathy, lagophthalmos, eyelid malposition, and unsatisfactory aesthetic outcomes [48-52]. Advanced imaging modalities, particularly computed tomography, were shown to improve the detection of associated orbital fractures, retained foreign bodies, and deep periocular tissue injuries in complex trauma cases [53-56].

One of the most consistent and clinically significant findings across the included studies was the clear benefit of early surgical intervention in eyelid trauma management. Repair performed within the first 24-48 hours after injury was consistently associated with lower postoperative complication rates, improved anatomical restoration, and superior functional and cosmetic outcomes [57-63]. Early intervention facilitates preservation of tissue vascularity, minimises oedema and fibrosis, and allows more accurate realignment of delicate periocular structures. In contrast, delayed reconstruction was frequently associated with increased tissue contracture, fibrosis, distorted wound architecture, infection risk, and greater technical difficulty during surgical repair, often resulting in suboptimal functional and aesthetic outcomes [64-67]. These observations strongly reinforce the fundamental principles of periocular reconstruction, which emphasise meticulous restoration of the eyelid margin, tarsal plate, canthal tendons, and lacrimal drainage system to maintain normal eyelid biomechanics, ocular surface protection, and tear film stability [68-72].

Canalicular injuries represented a major subset of periocular trauma cases within the reviewed literature. Early microsurgical repair with silicone intubation or stenting demonstrated high rates of anatomical patency and satisfactory lacrimal drainage outcomes [73-77]. Nevertheless, epiphora remained one of the most commonly reported postoperative complications, particularly in patients with delayed reconstruction or severe medial canthal injury [78-81]. These findings further reinforce the importance of meticulous exploration of all medial eyelid lacerations and prompt reconstruction of the lacrimal drainage system whenever canalicular involvement is suspected [82-85].

Complex eyelid defects involving avulsion injuries, full-thickness tissue loss, and burn-related trauma continue to pose major reconstructive challenges. Several studies have demonstrated favourable outcomes with advanced reconstructive procedures, including the Hughes tarsoconjunctival flap, Cutler-Beard technique, rotational flaps, composite grafts, and staged periocular reconstruction [86-90]. Importantly, successful eyelid reconstruction extends beyond tissue closure and requires restoration of eyelid mobility, blink function, ocular surface protection, and acceptable facial aesthetics [91-94]. Contemporary reconstructive algorithms and individualised flap-based techniques have further improved surgical planning and postoperative rehabilitation in patients with extensive periocular tissue loss [95-98].

Recent advances in oculoplastic and reconstructive surgery have expanded the therapeutic options available for complex eyelid trauma management. Emerging evidence supports the integration of minimally invasive procedures, modern flap advancement techniques, plasma-based tissue remodelling technologies, and advanced reconstructive biomaterials to improve wound healing and reduce postoperative morbidity [99-103]. Furthermore, recent systematic reviews evaluating reconstructive outcomes have emphasised the importance of individualised surgical planning, multidisciplinary collaboration, and evidence-based periocular reconstruction in optimising long-term outcomes [104-107].

The majority of included studies reported favourable postoperative outcomes, particularly when early repair and accurate anatomical reconstruction were achieved [108-110]. However, postoperative complications remained clinically significant in delayed or severe trauma cases. Epiphora, ectropion, entropion, eyelid retraction, scarring, infection, wound dehiscence, and persistent asymmetry were among the most frequently reported complications [57-63]. Patients with associated orbital fractures or globe injuries generally experienced poorer outcomes because of increased injury complexity and higher rates of secondary reconstructive procedures [111].

This systematic review possesses several strengths, including a comprehensive evaluation of contemporary literature, inclusion of geographically diverse patient populations, and a detailed assessment of reconstructive strategies and postoperative outcomes. Nevertheless, several limitations should be acknowledged. Considerable heterogeneity existed among the included studies regarding study design, patient populations, injury severity, outcome definitions, and follow-up duration. Additionally, the predominance of retrospective observational studies limits the overall level of evidence. Variability in reporting standards and reconstructive techniques also restricted the ability to perform extensive pooled quantitative analyses for certain outcomes [112,113].

Emerging trends and future directions

Artificial intelligence (AI), advanced imaging, and precision reconstructive technologies are increasingly transforming the management of eyelid trauma [96,103,105,109]. AI-driven diagnostic systems and machine learning algorithms have shown considerable potential in improving the early detection and assessment of periocular injuries through automated image analysis, pattern recognition, and quantitative evaluation of eyelid morphology [105,109]. These technologies may assist clinicians in identifying subtle anatomical abnormalities, predicting injury severity, and optimising reconstructive planning with greater accuracy and reproducibility.

AI-assisted predictive models are also emerging as valuable tools for prognostication and surgical decision-making. By integrating clinical findings, radiological imaging, and perioperative variables, machine learning systems may help estimate the risk of postoperative complications, functional impairment, and the need for secondary reconstructive procedures [96,105]. In addition, advanced imaging modalities, including three-dimensional (3D) periocular imaging and digital surgical mapping, have improved preoperative assessment by enabling precise evaluation of tissue loss, orbital involvement, eyelid contour abnormalities, and facial symmetry [93,100,103].

Recent developments in minimally invasive oculoplastic techniques, plasma-based tissue remodelling, regenerative biomaterials, and customised reconstructive approaches have further expanded therapeutic options for managing complex eyelid trauma [100,105,109]. These evolving technologies may contribute to reduced postoperative scarring, improved wound healing, enhanced tissue integration, and superior cosmetic rehabilitation. Furthermore, AI-assisted outcome analysis and digital morphometric assessment provide a more objective evaluation of postoperative eyelid position, symmetry, and functional recovery than conventional subjective clinical assessment methods.

Despite these promising advances, several important challenges continue to limit widespread clinical implementation. Current evidence remains limited by the lack of large-scale prospective validation studies, the absence of standardised AI protocols, concerns regarding data privacy and algorithmic bias, and difficulties integrating these technologies into routine clinical workflows [96,105,109]. Additionally, the cost and accessibility of advanced digital systems may restrict their application in low-resource healthcare settings.

Nevertheless, the integration of AI-driven diagnostics, advanced imaging, predictive analytics, and precision reconstructive surgery with multidisciplinary oculoplastic care is expected to significantly improve the future management of eyelid trauma. Continued technological innovation and evidence-based validation may ultimately enable more personalised, efficient, and outcome-oriented approaches to periocular trauma reconstruction [103,105,109].

Strengths and limitations

This systematic review provides a comprehensive and up-to-date synthesis of the current evidence on eyelid trauma management, incorporating 80 studies from diverse populations, geographic regions, and surgical settings. A major strength of this review is the inclusion of various study designs, including retrospective and prospective studies, systematic reviews, epidemiological analyses, and reconstructive case series, which enhances the overall clinical relevance and breadth of the findings. Consistent evidence across studies supports the importance of early intervention, precise anatomical reconstruction, canalicular preservation, and multidisciplinary management in achieving favourable functional and cosmetic outcomes. The review also highlights recent advances in oculoplastic reconstruction, including flap-based techniques, minimally invasive procedures, advanced imaging, and technology-assisted surgical planning, providing insight into evolving trends in periocular trauma care.

However, several limitations should be acknowledged. Most included studies were retrospective and observational, introducing potential selection and reporting biases. Significant heterogeneity existed regarding injury severity, patient characteristics, surgical techniques, outcome measures, and follow-up duration, limiting direct comparison and extensive quantitative synthesis. In addition, variability in surgeon expertise and healthcare infrastructure may affect the generalisability of findings. Long-term functional outcomes and patient-reported satisfaction were reported inconsistently, and evidence on emerging technologies such as AI and advanced imaging remains limited. Therefore, further high-quality prospective studies and standardised multicentre research are needed to strengthen the evidence base and optimise management algorithms for eyelid trauma.

Clinical implications

The findings of this systematic review strongly reinforce the critical importance of early diagnosis and timely surgical intervention in achieving optimal outcomes following eyelid trauma. Prompt recognition and management of associated injuries involving the canalicular system, globe, orbit, and canthal tendons are essential to minimise long-term morbidity, preserve ocular function, and optimise visual rehabilitation. Consistent evidence from the included studies demonstrates that early anatomical restoration significantly reduces postoperative complications and improves both functional recovery and cosmetic outcomes. The review further highlights the importance of individualised, defect-specific reconstructive strategies tailored to the extent of tissue loss, the complexity of the injury, and associated periocular involvement. Precise restoration of eyelid anatomy, preservation of eyelid biomechanics, maintenance of lacrimal drainage function, and protection of the ocular surface remain fundamental principles of successful reconstruction. In patients with complex periocular trauma, multidisciplinary collaboration among ophthalmologists, oculoplastic surgeons, plastic surgeons, and maxillofacial specialists is crucial for comprehensive assessment, coordinated management, and optimal surgical planning. Collectively, the evidence synthesised in this review supports a patient-centred, multidisciplinary, anatomically precise, and time-sensitive approach as the cornerstone of contemporary eyelid trauma management, with the potential to significantly improve long-term functional and aesthetic rehabilitation.

## Conclusions

This systematic review provides a comprehensive synthesis of contemporary evidence on the management of eyelid trauma, highlighting significant advancements in diagnostic evaluation, surgical techniques, and reconstructive strategies over the past two decades. The findings consistently demonstrate that early intervention, meticulous anatomical reconstruction, and individualised treatment planning are fundamental to achieving optimal functional and aesthetic outcomes. Primary layered repair remains the cornerstone of management for uncomplicated injuries, while advanced reconstructive techniques have expanded the therapeutic possibilities for complex defects. The integration of multidisciplinary care, particularly in cases involving orbital or globe injuries, further enhances clinical outcomes and reduces complication rates. Despite these advances, postoperative complications, such as epiphora and eyelid malposition, remain clinically relevant, especially in delayed or inadequately managed cases. Importantly, this review underscores the critical role of the timing of intervention as a modifiable factor that significantly influences prognosis. Emerging innovations, including minimally invasive techniques, biomaterials, and AI, hold promise for further improving precision and outcomes in eyelid trauma management.

However, the current evidence base is limited by heterogeneity in study design, variability in outcome reporting, and a predominance of retrospective studies, emphasising the need for standardised outcome measures and high-quality prospective research. In conclusion, optimal management of eyelid trauma requires a structured, time-sensitive, and multidisciplinary approach, with emphasis on early repair and precise surgical execution. Future research should focus on refining reconstructive algorithms and integrating emerging technologies to further enhance patient outcomes in this complex and evolving field of ophthalmology.
